# UNVEILING THE ASSOCIATION BETWEEN SOCIAL ISOLATION AND SLEEP DISTURBANCES AMONG LATINX OLDER WOMEN AND MEN

**DOI:** 10.1093/geroni/igae098.3762

**Published:** 2024-12-31

**Authors:** Luciana Giorgio, Erick Luz da Scherf, Nicole Ruggiano

**Affiliations:** University of Alabama School of Social Work, Tuscaloosa, Alabama, United States; University of Alabama, Tuscaloosa, Alabama, United States; University of Alabama, Tuscaloosa, Alabama, United States

## Abstract

**Background:**

Sleep disturbances are associated with an increased risk of cardiovascular, cardiometabolic, and neurodegenerative conditions. Aging involves physiologic changes associated with increased sleep disturbances, however, sociocultural stressors, such as social isolation, can exacerbate these disturbances. Although Latinx older adults are more likely to experience social isolation and poorer sleep than their non-Latinx White counterparts, they remain underrepresented in sleep health research. We examined the association between social isolation and sleep disturbances and explored gender differences in this relationship.

**Methods:**

We used data from Latinx older adults participating in the Hispanic Community Health Study/Study of Latinos Sociocultural Ancillary Study (n=323). We conducted weighted linear regression analyses adjusted for sociodemographics and selected health conditions to examine the relationship between social isolation and sleep disturbances. Sleep disturbances were measured using the Women’s Health Initiative Insomnia Rating Scale(WHIIRS). Social isolation was assessed using validated loneliness and social support scales. Gender differences were examined using cross-products.

**Results:**

Most participants were women (54.30%), immigrants (97.56%), Cuban (40.33%), and Spanish-speaking (86.00%). Participants reported mean loneliness of 1.44(SD=1.66) and social support of 26.40(SD=6.68). Average WHIIRS scores were 7.53(SD=5.68). Increasing loneliness was associated with a 0.48-point increase in WHIIRS (β=0.48, SE=0.24, p=0.04) in adjusted models. Gender was not a significant moderator in this relationship. Social support was not significantly associated with WHIIRS.

**Conclusions:**

Loneliness was a robust predictor of sleep disturbances among Latinx older adults. Psychological and community-level interventions aimed at increasing social connection may improve sleep, thus reducing the burden of health conditions in this population.

